# Analysis of motor behavior in piano performance from the mixed methods approach

**DOI:** 10.3389/fpsyg.2024.1433441

**Published:** 2024-09-05

**Authors:** Isabel E. Santisteban, M. Teresa Anguera, Juan Granda-Vera, José Luis Pastrana-Brincones

**Affiliations:** ^1^Faculty of Psychology, University of Barcelona, Barcelona, Spain; ^2^Faculty of Psychology, Institute of Neurosciences, University of Barcelona, Barcelona, Spain; ^3^Department of Didactics of Musical, Artistic and Corporal Expression, Faculty of Education and Sport Sciences Melill, University of Granada, Granada, Spain; ^4^Department of Languages and Computer Sciences, School of Computer Science and Engineering, University of Málaga, Málaga, Spain; ^5^Computer and Information Sciences, University of St. Thomas, St. Paul, MN, United States

**Keywords:** piano performance, mixed methods, observational methodology, pressed touch, struck touch

## Abstract

**Introduction:**

The focus of this study centers on the extraction, analysis, and interpretation of the motor behavior of advanced-level pianists using observational methodology, itself framed within the field of mixed methods, paying particular attention to those aspects that characterize the pressed and struck touch. The aim of this research was to analyze the motor interactions of activation or inhibition associated with the production of a type of touch in the movements of the right upper limb of the participating pianists.

**Methods:**

An *ad hoc* observational instrument was built that was incorporated into the software Lince Plus for data recording and coding. Data reliability was guaranteed applying Cohen’s Kappa coefficient, and an analysis of polar coordinates was carried out to identify the motor interactions involved in piano playing.

**Results:**

The study provided significant information about the interaction of motor functions linked to two types of touch, such as those that occur in the sliding finger movement over the key in the pressed touch or the lifting finger movement above the key in the struck touch, obtaining clearly identified patterns of piano touch motor behavior.

**Discussion:**

This research represents an innovative perspective of the study of piano-playing movement via the direct and perceptible observation of the pianist’s motor behavior in an everyday context. Observational methodology is distinguished by its low degree of internal control, which makes it possible to scientifically study the spontaneous behavior of pianists in their natural environment. This model allows us to describe and analyze piano touch for its application in the field of piano performance and teaching, emphasizing the practical implications of motor interactions in piano touch.

## 1 Introduction

The study of piano touch is an essential aspect of piano playing, as much on a pedagogical level as on a biomechanical, technical, and, above all, artistic level. The sound and expressive intentions of the pianist are reflected on the keyboard through motor actions of the upper limbs. The control of the quality and precision of piano playing is acquired over many years of practice and study, geared towards perfecting the pianist’s motor abilities (Furuya et al., 2010; Goebl et al., 2005). The studies of Furuya et al. (2014a, 2014b) highlighted optimized finger movements and a higher independence of these, achieved via musical practice, suggesting a plastic adaptation of the neuromuscular system associated with the control and independence of finger movement. Similarly, the pianist can specify the sound quality—defined from a timbral viewpoint—with gesture and type of touch (Bernays and Traube, 2013; Dogantan-Dack, 2016). In MacRitchie’s multi-disciplinary review of piano touch (2015), it was noted that, in addition to the different individual characteristics of each pianist, the sound of the piano also differed depending on the technical resources used, with type of touch—pressed versus struck—playing a significant role. Goebl et al. (2005) analyzed these two types of touch, observing large differences in the depression of the key and in hammer speed patterns. They examined the contact times of the whole set of pieces that make up the percussion mechanism of the piano, highlighting the importance of touch in movement control. They showed that, at the same speed, there is greater sound control with the pressed touch than the struck touch, with manipulation of the instrument and playing technique influencing perception of the sound quality of the playing. Furthermore, the pianist’s movements are a relevant factor in the effective communication of the musician with the audience (James, 2018; Li, 2022).

Advances in the scientific research of piano touch have revealed that these two types of touch exhibit different motor behaviors of the upper limbs (Goebl et al., 2014; Furuya et al., 2010) that imply different muscular loads, along with different sound qualities. The integration of both types of touch is suggested, although highlighting that a staccato articulation would carry a higher risk of injury, above all in the shoulder (Degrave et al., 2020; Goebl et al., 2004).

MacRitchie and Zicari (2012) highlighted the importance of the connection between the pianist’s musical intention and the resulting physical gesture, concluding that the musician’s conscious control over their body influences the sound produced.

The timbral quality of a performance, from the pianists’ viewpoint, does not only depend on the intensity and duration of the sounds, but includes various aspects that make up the performance: melody, articulation, tempo or dynamics. They use adjectives and metaphors such as shiny, rounded, dry, thick or velvety to describe different timbral nuances (Bernays, 2013; Li and Timmers, 2021). The embodiment of these musical conceptions requires the interaction between the plasticity of the neuronal system and the motor system, characterized by its flexibility in organizing an inordinate number of degrees of freedom in the upper limbs (Bernstein, 1967; Kay et al., 2003; Furuya and Altenmüller, 2013).

In the last few decades there has been a significant increase in research into acoustics and biomechanics in the field of piano playing. The majority of these studies use technical measuring systems equipped with high precision sensors attached to both the pianist’s body and the instrument itself (Goebl, 2017). These studies have provided valuable scientific information that sheds light on the artistic area of piano technique, and specifically—in the case that concerns us—on the knowledge of piano touch procedures. See the research of MacRitchie and McPherson (2015), Bernays and Traube (2014) or Turner et al. (2021), among others, as clarifying examples.

However, it remains a challenge to study the pianist’s body movements in a natural and minimally intrusive environment that does not significantly affect their performance (Goebl, 2017). As MacRitchie (2015) points out, there is a need to develop non-intrusive measurement systems that can be incorporated into piano lessons, outside of a laboratory setting and without restrictions.

The application of observational methodology to this approach is considered appropriate, since it is characterized as a scientific method adapted to the reality of natural situations, in which the control of other methodologies is not suitable, given that it requires the spontaneity of human behavior developed in its usual context (Anguera et al., 2019). Until now, observational methodology has been primarily applied to the study of movement in motor contexts such as physical activity and sports (Anguera et al., 2017). However, it has not been used in the analysis of body movement in the field of instrumental music.

This study seeks to fill this gap in the scientific literature related to this subject by applying observational methodology to the study of piano-playing movement which contributes—in addition to a non-intrusive method of capturing movement—a new analysis of the behavioral flow of piano touch that implies an order of motor actions. Lag sequential analysis and polar coordinate analysis appear as a powerful medium for estimating patterns of behavior that reflect activation and inhibition relationships of movements that precede or follow a particular behavior, in such a way that they reflect behavioral tendencies that can be useful for detecting motor behavior patterns that could be avoided in order to prevent injury or perfect both technique and musical expression. Lag sequential analysis of piano performance is different from other purely quantitative analyses due to its implication in the process of transition and interconnection between some behaviors and others, involving a continual systematic observation process that takes into account the technical and expressive requirements of the musical piece. This analysis can provide a deeper understanding of the piano touch technique.

This project puts forward an innovative approach to the study of the motor behavior of two advanced level pianists, using observational methodology (OM)—considered mixed method in itself—(Anguera et al., 2017, 2018b; Anguera and Hernández-Mendo, 2016), for its adaptability and flexibility in detecting and analyzing, via direct and perceptible observation, the motor behavior patterns that are shown in the expressive production of piano sound in natural contexts. OM integrates the qualitative and quantitative phases (QUAL-QUAN-QUAL) of the research via rigorous procedures (quantitizing) that render it robust by making data analysis possible, not only through the scrutiny of behavior occurrences with the frequency parameter, but with the parameters of order and duration, from the production of a data record in the form of a code matrix (Anguera et al., 2020).

The main aim of this research was to identify relationships between motor behaviors that differently characterize pressed and struck touch. To this end we present and justify the construction of an *ad hoc* observation instrument based on field format, for its open, multidimensional, multiple-code and self-regulatory character (Anguera, 2010), integrated into the software LINCE PLUS for systematic observational study (Soto-Fernández et al., 2022).

The observation produced a large set of qualitative data that was analyzed quantitatively via lag sequential analysis (Bakeman and Gottman, 1989; Bakeman and Quera, 2011) and polar coordinate analysis (Sackett, 1980). This analysis yielded novel results regarding the detection of motor interactions linked to piano-playing movement.

## 2 Pressed touch and struck touch

The study of piano touch covers diverse aspects related to motor behavior, such as the movement of fingers, hands, upper arms and forearms, wrists and shoulders, along with speed or the pressure applied to the keys, analyzed from different pedagogical, biomechanical and technological perspectives. Type of touch is a highly important procedural parameter, given that it produces different sound and expression qualities that are perceived both by the musician and the audience (Goebl, 2017). Pianists are unanimous in distinguishing between pressed and struck touch, with the different motor behaviors that each one generates (Goebl et al., 2014; Furuya et al., 2010) and the repercussion in the quality—both of sound and expression—in piano playing (Goebl et al., 2005). The pressed touch is initiated from contact with the surface of the key, increasing shoulder mobility during the anticipatory rocking movement of pressing the keys (Verdugo et al., 2020, 2022); while the struck touch is characterized by lifting the finger a certain distance from the key before pressing it, producing an additional noise in the piano sound (Goebl et al., 2005; Kinoshita et al., 2007). Furuya et al. (2010) described a sound with a “hard” timbre if the key is pressed after previously lifting the finger and a “soft” timbre if the keystroke starts with the finger in contact with the key. Similarly, Goebl et al. (2014) showed that the timbre and the sound quality of the piano are not only influenced by the speed with which the hammer reaches the string. It was shown that applying the same speed to the keys, but with different touches, it was possible to detect the noise made by the finger-key and key-bottom impact if struck touch was used, which did not happen with the pressed touch. On the other hand, with the pressed touch, the sound reflects a gradual increase in the key speed, while the struck sound involves a sudden initial peak. With the struck touch, the time the finger stays on the key is shorter given that the finger hits the key at speed and leaves it quickly; while in the pressed touch the finger leaves the key by sliding off it in gradual acceleration, remaining on the key longer (Ortmann, 1925; W, 1925) and thus lengthening the acoustic qualities of the piano. While the struck touch is ineffective for this purpose, it is effective for strong sounds and rapid movements (Goebl et al., 2014). In terms of finger movement during the keystroke, Furuya et al. (2010) compared the pressed touch with the struck touch, finding that in the former the finger traces a trajectory from the distal phalanx (DP) to the proximal phalanx (PP), while in the latter it was from the PP to the DP; thus a gentler sound is produced—from a psychoacoustic perspective—in the pressed touch than in the struck touch. The trajectory of the pressed touch is therefore closer to the *carezzando* style of Chopin and the Lisztian touch, characterized by pressing the key with the finger and releasing it by sliding over it from the fingerpad towards the palm of the hand (Bellman, 2001; Ott, 2003).

James (2012) considers the use of the pressed touch essential for obtaining tonal quality; this is because the contact surface area of the fleshy part of the finger is larger, due to the fingers being slightly extended, providing the musician with greater tactile feedback. Regarding the position of the fingers, Furuya et al. (2012) also concluded that in the pressed touch the fingers are more extended than curved, facilitating a larger surface contact. In this sense, Goebl and Palmer (2008) highlighted that the mechanoreceptors in the skin of the fingerpad transmit sensorial signals to large cortical areas responsible for tactile processing, with the proximity of the fingers to the keys being significant (Dalla Bella and Palmer, 2011). This is essential for the precision and spatial exactitude necessary for controlling the speed of contact with the key, the articulation and *tempo*, together with obtaining different tonal nuances (James, 2012; Kojucharov, 2014; Kojucharov and Rodà, 2015). According to the pianist, pedagogue and precursor of the scientific study of piano touch, Marie Jaëll, the fingerpad touch enables the musician to obtain an ideal sound via a system of reciprocal influence of movement, thought and internal hearing (Robb, 2022).

With reference to the pianist’s control of the touch, Goebl and Palmer (2008) observed that this depends on the parameters of *tempo* and dynamics, with the struck touch being more common in faster *tempi*, whilst in slows *tempi* pianists have greater control over the type of touch. Furthermore, use of the pressed touch stands out in gentle dynamics, whilst the struck touch is preferred in stronger dynamics (Goebl et al., 2005). In their study of time control and the efficiency of expert musicians’ hand movements, Goebl and Palmer (2013) observed that the fingers act independently at different *tempi*. They also observed that wrist movements of supination, pronation, flexion and extension remained stable in different *tempi*, as opposed to the finger joints which had greater ranges of motion. Hadjakos et al. (2008) highlighted shoulder and elbow participation—together with wrist and finger joints—in octave repetition, the forearm being lifted with inertia to avoid tiredness during the pianist’s performance. In terms of wrist action, Oikawa et al. (2011) observed that the flexor muscle in the wrist is what acts as an agonist in the movement, with rapid activation to press the key, while the extensor stabilizes the wrist and finger joints. Using surface EMG, they concluded that a neutral wrist position at *fortissimo* intensity is the best option for reducing the musculoskeletal load on the forearm during the keystroke. The height of the finger when preparing to press the key also varied with changes in rhythm and dynamics (Dalla Bella and Palmer, 2011). One of the most important aspects of the keystroke is the movement and shape of the fingers. Among the aspects that influence the keystroke, Berman (2002) included: the weight placed on the key, the mass and speed applied, the comparison between the finger that reaches the key bottom and that which superficially touches it, the curve of the fingers and the contact applied to the key by the finger pad or tip; along with the position inside or outside the keyboard with pressure on the keys or remaining at a distance without a strong grip on them. Far from being of little relevance, these characteristics define the timbre quality of a performance and distinguish one pianist from the next (Sandor, 1981). Regarding the shape of the palm area of the hand, Brown (2000) suggested that flattening the arch of the hand at the point of the knuckles creates tension in the forearm and fingers. Following this logic, Del Pueyo (1990) maintains that the hand should be reinforced and balanced with firm fingers, forming a hollow by the marked lowering of the outer fingers. With the purpose of avoiding blocking the hand, Lee (2005) makes reference to the piano technique of Franz Liszt, which involves the suspension and sliding of the fingers—just like the bow of a violin when it falls onto the strings and slides over them—lightening the weight to enable a wide expression of different dynamics, thanks to retropulsive force characterized, according to Ott (2004), as:

A light suspension of the shoulder and upper arm that allows the forearm to tilt its pressure towards the hand while a large fleshy surface of the fingers pulls on the keys, retaining this general retropulsion at the key bottom which provides strength and speed without causing any blocking (p. 37).

The hand no longer stays still but begins to move freely to the right and left, excluding verticality, highlighting the role of the finger flexors for this purpose. Uniformity in the fingers is achieved through gradually supporting the forearm and upper arm, avoiding the heaviness produced by the force of gravity (Ott, 2004). In his treatise about piano playing technique, *On piano playing: motion, sound and expression,* Sandor (1981) explains free-fall movement: consisting of the upper arm, forearm, hand and fingers lifting and then falling using gravitational force, with no muscular intervention whatsoever during the lowering movement of the arms. When the fingers reach the keyboard a slight elevation of the hand is produced while the fingers remain over the keyboard. On the other hand, regarding the complex motor behavior of piano touch, Bernstein (1967) and Bernstein and Popova (Kay et al., 2003), showed how muscle strengths were reorganized from a biomechanical viewpoint depending on the *tempi*, in such a way that the forearm participated in a slow *tempo*, whilst only the wrist flexion intervened in a rapid *tempo.* This showed the motor system’s capacity for self-organization contingent on mechanical restrictions, along with the possibility that different movements produce similar results (Kursell, 2006). Furuya and Kinoshita (2008) evidenced the importance of training for expert pianists, in their study of motor control of the upper limbs in the interaction of complex multi-joint movement—shoulder, arm, elbow, wrist and metacarpophalangeal joints—during piano touch. Such training is fundamental for achieving greater physiological efficiency, along with a greater exactitude of movement, which implies a necessary muscular control of the upper limbs as a whole. The precursor of scientific research into the piano touch mechanism, Marie Jaëll, in her empirical study of hand movement (Caruso, 2016), considered that musical perfection is achieved through a corporal awareness of piano playing, avoiding the automatic repetition of movements (Guichard, 2004). For Jaëll, isolation and paying attention to minimal variations in touch is similar to tuning in to the aesthetic content of a musical work (Weinstein-Reiman, 2021). Emphasizing this aspect, it is important to underline the importance of conscious activity in order to intervene in muscular counter-tension that counteracts the motion impulse of the arm, hand and fingers. This is achieved via a continuous movement that enables muscular control of finger rebound on the keys, acting in the opposite direction to this, using tension in opposition to the force of gravity that develops muscular elasticity starting from points of support (James, 2012). In short, the pianist’s body movements are related to the control of the kinematic chain from the shoulders to the finger pads, combining speed—according to the *tempo* and dynamics—with musical expression (Goebl, 2017).

## 3 Methods

### 3.1 Design

A mixed method approach was applied to this study using observational methodology (Anguera, 1979). Following the three dichotomous dimensions proffered by Anguera et al. (2011), the design proposed in this research was of an N/P/M type (nomothetic in being aimed at the observation of each of the two participants and the independent study of them being of interest; punctual, as the recordings were carried out in one single session and in the same conditions for both participants although with intra-session monitoring from the start to the end of the session; and multidimensional, since different behaviors are observed which entail different levels of response, with corresponding repercussion in the observation instrument). The systematic observation carried out was non-participatory and the observed components were completely perceptible (Anguera et al., 2018a; Bakeman and Quera, 2011).

### 3.2 Participants

The participants in this study were two male pianists aged 28 and 27, identified as Participant 1 (P1) and Participant 2 (P2), respectively. Both had advanced qualifications in the specialty of piano, with 21 and 20 years of professional experience, respectively, in the study of classical piano.

Experienced pianists were required with a view to maximizing the degree of control in the sound production in their performance. Both musicians gave their written informed consent in accordance with the principals of the Helsinki Declaration, and a favorable assessment was obtained of the methodological, ethical and legal aspects from the Bioethical Commission of the University of Barcelona.

### 3.3 Instruments

#### 3.3.1 Observation instrument

An *ad hoc* observation instrument was constructed for the observation and record of the motor behaviors of the participants during the piano playing performance. The aim was to detect, select and analyze patterns of motor behavior in the piano touch of each of the two musicians. As it is a multidimensional study, the observation instrument was based on the field format, which contained 13 dimensions deployed in 53 behaviors in total, the mutual exclusivity of these behaviors being governed in each of the dimensions (Table 1). The base dimension for the construction of the observation instrument were the motor functions of the right upper limb in piano playing, under the technical premises of the struck touch and the pressed touch. The segmentation criteria were based on selecting units of behavior according to the technical requirements of the musical excerpt performed, taking into account the specific purposes of the research, which focus on observing the piano touch of two expert pianists during the performance. Nine behavioral segments were delimited after the exploratory phase of the study. These are shown in Dimension 1 of the observation instrument (Table 1), with the molar and molecular levels of the behaviors observed, taking into account the different levels of granularity (Schegloff, 2000) of the units—from larger (molar level) to smaller (molecular level) –, and considering their relative nature, included within the dimensions and behaviors of the observation instrument.

**Table 1:** Observational instrument.

Dimensions	Dimension code	Behaviors/elements	Behaviors/elements description	Behaviors/elements code
1. Musical extract performed	ExMu	1st demarcation Mozart Sonata N° 13 in B-flat major	Moment immediately prior to the start of the performance.	Mzt1_1
		2nd demarcation Mozart Sonata N° 13 in B-flat major	Playing of the first note of the beginning of the Sonata, note “G,” in the attack and release of the key. 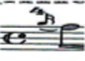	Mzt1_2
		3^rd^ demarcation Mozart Sonata N° 13 in B-flat major	Beginning of the Sonata until the second beat of the first bar, except the already analyzed first note “G.” Descending succession of sounds by consecutive degrees. 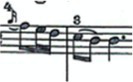	Mzt1_3
		4th demarcation Mozart Sonata N° 13 in B-flat major	2nd bar: appoggiatura on the note “F” and note “E-flat”/“G” semiquaver of the third beat of the 2nd bar and movement from “C” at the end of the 2nd bar to the 3rd bar with appoggiatura in “B-flat” of the first beat of the bar. 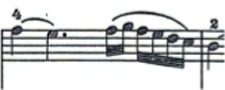	Mzt1_4
		5th demarcation Mozart Sonata N° 13 in B-flat major	4th bar: appoggiatura of double notes at a distance of a 3rd interval of the first beat (A-C) and double notes in a 3rd interval of the second beat of the bar (B-flat-D). 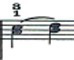	Mzt1_5
		6th demarcation Mozart Sonata N° 13 in B-flat major	Movement from 4th to 5th bar. 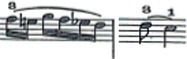	Mzt1_6
		7th demarcation Mozart Sonata N° 13 in B-flat major	5th bar: syncopation of the third beat of the bar: “D-C-A.” 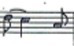	Mzt1_7
		8th demarcation Mozart Sonata N° 13 in B-flat major	7th bar: 3rd and 4th beat (appoggiaturas), and “B-flat” of the 8th bar. 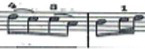	Mzt1_8
		9th demarcation Mozart Sonata N° 13 in B-flat major	Whole of 9th bar and two first beats of the 10th bar: Syncopation and descending succession of sixths until the end of the first theme of the Sonata with the note “B-flat” of the second beat of the 10th bar. 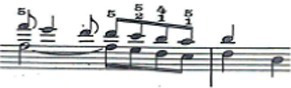	Mzt1_9
2. Start of keystroke	IPT	From contact with the key	The finger initiates the keystroke from the surface contact with the key.	Dcontsup
		Lifts finger before contact with the key	The finger lifts before contact, with extension of the metacarpophalangeal joint.	DelvextM
		Lifts the arm before the keystroke	Arm lifted prior to the keystroke.	Levprevbz
3. Fingers	DS	M flexion and slight P and D extension	With flexion of the metacarpophalangeal joint and slight extension of the proximal and distal interphalangeal joints.	DfxMligextPD
		M, P and D Flexion	With flexion of the metacarpophalangeal, proximal and distal joints.	DfxMPD
		M flexion and P and D extension	With flexion of the metacarpophalangeal joint and extension of the proximal and distal interphalangeal joints.	DfxMextPD
		M extension and P and D flexion	With extension of the metacarpophalangeal joint and flexion of the proximal and distal interphalangeal joints.	DextMfxPD
		M, P and D extension	With extension of the metacarpophalangeal, distal and proximal joints.	DextMPD
4. Fingers: surface contact with the key	DSct	Fingerpad	Makes contact via the fingerpad.	Dcy
		Distal phalanx	Makes contact by resting the distal phalanx on the key.	DcfgD
		Distal and proximal phalanx	Makes contact by resting the distal and proximal phalanx on the key.	DcfgDP
		Fingertip	Makes contact with the fingertip.	Dcpt
		Lateral in supination	Makes contact with the lateral part of the finger rotating in supination.	Dcrsup
		Lateral in pronation	Makes contact with the lateral part of the finger rotating in pronation.	Dcrpro
		No contact	No contact with the key.	DNoc
5. Thumb: surface contact	PScT	Lateral	Makes contact via the lateral part of the thumb.	PcL
		Fingerpad	Makes contact via the fingerpad with extension of the interphalangeal thumb joint.	PcY
		Fingertip	Makes contact via the fingertip with flexion of the interphalangeal thumb joint.	PcP
		No contact	Makes no contact with the key.	PNoc
6. Finger: key release	DLibT	Sliding over the key	The finger releases the key by sliding over it towards the palm of the hand.	Ddz
		Lifting above the key	The finger releases the key by lifting from the point where contact is made in the moment of the keystroke.	Delv
7. Finger placement	DsCol	Similar	The fingers are placed in a similar way close to the surface of the keys.	DsSim
		Disparate	The fingers are placed in a disparate way: in contact with the key, separated from the key, curved or extended.	DsDisp
8. Thumb: movements	P	Thumb pass with forearm rotation	The thumb moves under the palm of the hand via rotation of the forearm and lifting of the elbow.	PpsR
		The thumb pivots to allow passage of other fingers	The thumb rotates on itself, acting as a pivot, at the same time that the other fingers pass over it. The hand rotates to change position.	PpivtPsDs
		Thumb slides to allow passage of other fingers.	The thumb slides over the key to facilitate the passage of the other fingers over it, with lateral hand displacement without change of position.	PdzPsDs
		Lateral thumb movement	The thumb moves laterally with the hand remaining in the same position, while the other fingers slide to let it through.	PdpzLt
9. Hand: palm area	MP	Cupped	Cupped palm area due to flexion of the metacarpophalangeal joints.	Moq
		Flat	Flat palm area due to extension of the metacarpophalangeal joints.	Mpl
10. Hand: action	Macc	Sliding movement	The fingers slide to facilitate movement of the hand over the keyboard without modifying its position.	MdlzD
		Movement with pronation turns	The hand moves over the keyboard turning in pronation, modifying its position.	Mgirpro
		Movement with supination turns	The hand moves over the keyboard turning in supination, modifying its position.	Mgirsup
		White keys area	The hand develops the action in the white keys zone.	MaccBlan
		Back of keyboard	The hand develops the action at the back of the keyboard.	MaccFond
		Away from the keyboard	The hand develops the action with the palm area away from the keyboard.	MaccFuer
		Jumping movement	The hand jumps over the keyboard.	MaccSalt
11. Wrist	Mca	Neutral position	Wrist in neutral position.	McaN
		Raised position	Wrist lifted via joint flexion.	McaF
		Low position	Wrist lowered via joint extension.	McaEx
12. Forearm	AtBz	High	Stays lifted sloping down toward the keyboard.	AtBzA
		Low	Stays lowered with the elbow facing the floor.	AtBzB
13. Placement of the body in front of the keyboard.	CC	High	Sits high up forming an obtuse angle between the upper arm and the forearm, with the forearm facing the keyboard.	CCa
		Low	Sits low down forming an acute angle between the upper arm and forearm.	CCb
		In line with the keyboard	Sits in line with the keyboard forming a right angle between the upper arm and forearm.	CCr

Dimension 1 (Musical extract interpreted) was deployed in nine behaviors/elements, each one containing musical extracts selected for observation, taking into account the technical requirements of piano playing, described textually and graphically in the observation instrument. Dimension 2 (Start of keystroke) was deployed in three behaviors related to finger or arm movement prior to the keystroke. Dimension 3 (Fingers) was deployed in five behaviors related to the different flexion or extension behaviors of the metacarpophalangeal joints (MJ) and the proximal (PJ) and distal interphalangeal finger joints (DJ). Dimension 4 (Fingers: surface contact with the key) was deployed in seven behaviors related to the part of the finger that made contact with the key (pad, tip, distal phalanx (DP), distal phalanx and proximal phalanx (DP and PP), lateral part in pronation or supination or no contact with the key). Dimension 5 (Thumb: contact surface) was deployed in four behaviors relating to the surface contact of the thumb with the key, taking into account the morphological difference of the thumb with respect to the other fingers. Dimension 6 (Finger: key release) was deployed in two behaviors, taking into account whether the finger releases the key by sliding over it or lifting it off the key. Dimension 7 (Finger placement), deployed in two behaviors, had the aim of observing the behavior of the fingers in terms of coordination or lack of coordination between them, taking into account similar or disparate placement of each finger with respect to the others. Dimension 8 (Thumb: movements) was deployed in four behaviors, taking into account the thumb movements via rotation or sliding over the key. Dimension 9 (Hand: palm area) was deployed in two behaviors, taking into account the cupped position of the palm, via flexion of the MJ, or the flat position, via extension of the MJ. Dimension 10 (Hand: action) was deployed in six behaviors, taking into account the hand movements or where the hand is placed on the keyboard when it is motionless. Dimension 11 (Wrist), was deployed in three behaviors according to the neutral, flexed or extended position of the wrist. Dimension 12 (Forearm) was deployed in two behaviors according to the position of the forearm in relation to the keyboard, being high when it is sloping down toward the keyboard and low when the elbow is facing the floor. Dimension 13 (Placement of the body in front of the keyboard) was deployed in three behaviors related to the height chosen by the pianist in front of the keyboard, according to the formation of an obtuse, acute or right angle by the upper arm and forearm. The dimensions and behaviors are shown in Table 1.

#### 3.3.2 Recording instruments

Filming was done using two Casio EXF-1 digital cameras which, prior to determining the system of reference, were placed on both sides of the participating pianists, at the height of the keyboard. The recording rate was 100 frames per second.

For the data record and coding process the program LINCE PLUS (v.2.1.0) (Soto-Fernández et al., 2022) was used. Bakeman (1978) established four types of observational data, and this classification is still valid. From these, we used Type IV data in this study, which require a multidimensional observation instrument, as is our case, and also that the duration of each co-occurrence of behaviors (in addition to their frequency and order of presentation) be recorded. They are the most powerful data and have allowed us to obtain a record with the maximum level of information, enabling us to then proceed to a robust analysis of that record. Type IV data are concurrent and time-based; i.e., they incorporate the parameters of order and duration, in addition to co-occurrence, given the multidimensional nature of the observation design.

#### 3.3.3 Audio recording instrument

Audio recording of the musical fragment performed by the participants was done with the software Ableton Live v.8, sound card Roland Edirol UA 101. The sound was recorded with no equalizing or added effects.

### 3.4 Procedure

**Figure 1:**
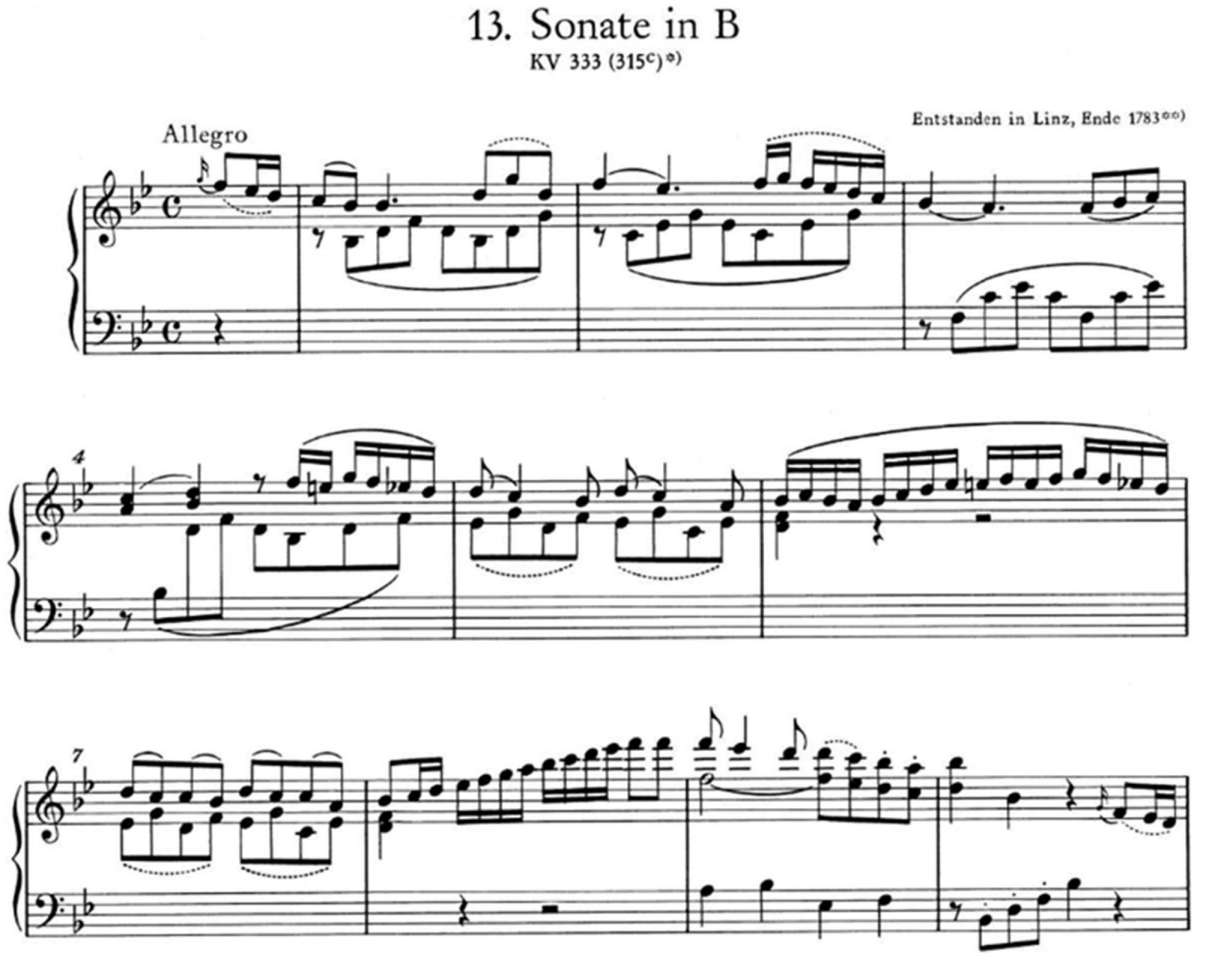
Excerpt of Sonata N° 13 for piano in B-flat major, K. 333/315c, by W. A. Mozart.

For this study a musical fragment was selected, consisting of the first 10 bars of the first movement of Sonata for N° 13 in B-flat major, K. 333/315c, by Wolfgang Amadeus Mozart (Figure 1). Eleven advanced level pianists were offered the opportunity to take part in the recording of this musical fragment, and their written informed consent sought. They were subsequently assessed by a group of three experts, two of these being teachers from a Spanish advanced music school, and the third a teacher from a Belgian music school. The selection was based on the evaluation of sound quality via a description using five adjectives considered by Bernays (2013) as the most common for describing nuances of timbre on the piano. Similarly, each of the audio recordings was given a score by the experts on a scale from 1 to 10. Finally, the two pianists who obtained the highest score from the experts were selected for this study (P1 and P2).

The participating pianists were asked to study the musical fragment for 5 min each day for a week. Following this practice time, the participating pianists were then recorded in both video and audio format playing the musical fragment on a Steinway grand piano placed center stage, in one single session which took place in the concert hall of the Auditorio Manuel de Falla in Granada, Spain. Each participant played the selected fragment with their own fingering, without using the pedal and in a *tempo* of 4 = 116. The recordings were carried out with two cameras placed on the sides of the piano keyboard, in such a way that the right upper limb study object could be observed from two different angles, to enable full inspection of it.

#### 3.4.1 Data quality control: inter-observer agreement

The record of the observed behaviors was done by two observers with a high degree of experience and knowledge of the substantive field of this study (Anguera et al., 2018a). One of them is a music teacher, with a qualification in piano teaching, an advanced qualification in dance and a qualification in the Dalcroze method based on the teaching of music through movement. The other observer is a professor of piano in an advanced music school and also a concert pianist. Both underwent 3 weeks of training in the observation process and use of the recording instrument for this study, prior to recording. Data record reliability was guaranteed by calculating the level of agreement with Cohen’s Kappa coefficient (Cohen, 1960, 1968), via the program Lince Plus, version 2.1.0 (Soto-Fernández et al., 2022). In line with Landis and Koch (1977), the inter-observer agreement level was “almost perfect,” obtaining results of 0.90 with participant P1, and 0.95 with participant P2.

#### 3.4.2 Data analysis

Once data reliability had been confirmed, the data were then analyzed. The robustness provided by observational methodology comes not only through the scrutiny of behavior occurrences with the first frequency parameter, but also because the parameters of order and duration enable lag sequential analysis and polar coordinate analysis, among others, thus rendering it necessary to obtain a data record in the form of a code matrix (Anguera et al., 2020).

Lag sequential analysis (Bakeman and Gottman, 1989; Bakeman and Quera, 2011), which was done from the obtained records, enables the detection of sequential patterns or chains of significant behaviors that show regularities in which patterns of motor behaviors can be observed, associated with a given behavior criterion, which is the behavior proposed in each analysis as the trigger of the behavioral pattern. The results are obtained in the form of adjusted residuals.

Polar coordinate analysis (Sackett, 1980), which requires as data the adjusted residuals obtained from the lag sequential analysis, is a powerful data reduction technique for determining the inhibition or activation relationships between a focal behavior (considered nuclear in each analysis) and one or various conditioned behaviors, which are all those of which we want to determine their activating or inhibiting relationship with the focal behavior. The associations between the behavior dyads are represented graphically via vectors.

Polar coordinate analysis was done in this study with the program HOISAN (Hernández-Mendo et al., 2012), version 2.0, with the observation record files being exported from LINCE PLUS to HOISAN.

As a preliminary step, lag sequential analysis was done to obtain prospective and retrospective patterns of behavior with respect to a given focal behavior. The lag sequential analyses were carried out prospectively (from +1 to +5 lags) and retrospectively (from −1 to −5 lags); the significance level was set at *p* < 0.05. The calculation of adjusted residuals gives positive (activation effect) or negative (inhibitive effect) values between the criterion behavior (that would play the role of focal in the polar coordinate analysis), and each conditioned behavior, which shows the degree of connection between both behaviors.

The volume of initial results produced was reduced via a powerful algorithm, based on the parameter Z_sum_ = ∑𝛧/√n, proposed by Cochran (1954) which is calculated prospectively (from the focal behavior onwards) and retrospectively (from the focal behavior backwards). This Z_sum_ parameter enables us to obtain the length and angle of the vectors that show the type of inter-relationship between the focal behavior and each conditioned behavior (depending on the quadrant they are in, and therefore the angle), and the intensity of this relationship (depending on the length of the vector).

In this study, polar coordinate analysis was used to analyze the focal and conditioned behaviors of the participants P1 and P2 that are shown in Table 2, in line with the aim of this study, where the codes corresponding to each behavior are indicated and the description recorded in the observation instrument (Table 1).

**Table 2:** Description and codes of the given focal behaviors and conditioned behaviors/elements of participants P1 and P2 in the polar coordinate analysis.

P1 Focal behaviors/elements	P1 Conditioned behaviors
Fingers with flexion of the metacarpophalangeal joint and slight extension of the proximal and distal interphalangeal joints. [DS_DfxMligextPD]	Start of keystroke_From contact with the key [IPT_Dcontsup]/_Lifts finger before contact with the key [IPT_ DelvextM]/_Arm lifted prior to the keystroke [IPT_Levprevbz]
	Fingers surface contact with the key_Fingerpad [DSct_Dcy]/_Distal phalanx [DSct_DcfgD]/_Distal and proximal phalanx [DSct_DcfgDP]/_No contact [DSct_DNoc]
	Finger key release_Sliding over the key [DLibT_Ddz]
	Finger placement_Similar [DsCol_DsSim]
	Hand: palm area_Cupped [MP_Moq]
	Wrist_Neutral position [Mca_McaN]/_Raised position [Mca_McaF]/_Low position [Mca_McaEx]
	Forearm_High [AtBz_AtBzA]
	Placement of the body in front of the keyboard_High [CC_CCa]
Fingers with flexion of the metacarpophalangeal, proximal and distal joints. [DS_DfxMPD]	Start of keystroke_From contact with the key [IPT_Dcontsup]/_Arm lifted prior to the keystroke [IPT_Levprevbz]
	Fingers surface contact with the key_Fingerpad [DSct_Dcy]/_Distal phalanx [DSct_DcfgD]/_Distal and proximal phalanx [DSct_DcfgDP]/_No contact [DSct_DNoc]
	Finger key release_Sliding over the key [DLibT_Ddz]
	Finger placement_Similar [DsCol_DsSim]
	Hand: palm area_Cupped [MP_Moq]
	Wrist_Neutral position [Mca_McaN]/_Raised position [Mca_McaF]/_Low position [Mca_McaEx]
	Forearm_ High [AtBz_AtBzA]
	Placement of the body in front of the keyboard_High [CC_CCa]
Thumb: surface contact via the lateral part of the thumb. [PScT_Pcl]	Thumb slides to allow passage of other fingers [P_PdzPsDs]/_Lateral thumb movement [P_dpzLt]
	Hand: palm area_Cupped [MP_Moq]
	Hand: action_Sliding movement [Macc_MdlzD]/_White keys area [Macc_MaccBlan]
	Wrist_Neutral position [Mca_McaN]/_Raised position [Mca_McaF]
	Forearm_High [AtBz_AtBzA]
	Placement of the body in front of the keyboard_High [CC_CCa]
9th demarcation Mozart Sonata N° 13 in B-flat major. [ExMu_Mzt1_9]	Start of keystroke_From contact with the key [IPT_Dcontsup]/_Arm lifted prior to the keystroke [IPT_Levprevbz]
	Fingers with flexion of the metacarpophalangeal joint and extension of the proximal and distal interphalangeal joints [DS_DfxMextPD]/_with extension of the metacarpophalangeal, distal and proximal joints [DS_DextMPD]
	Fingers surface contact with the key_Fingerpad [DSct_Dcy]/_Distal phalanx [DSct_DcfgD]/_Distal and proximal phalanx [DSct_DcfgDP]
	Thumb: surface contact via the lateral part of the thumb
	[PScT_Pcl]/_Fingertip [PScT_PcP]
	Finger key release_Sliding over the key [DLibT_Ddz]
	Finger placement_Similar [DsCol_DsSim]
	Hand: palm area_Cupped [MP_Moq]/_ Flat [MP_Mpl]
	Hand: action_Sliding movement [Macc_MdlzD]/_White keys area [Macc_MaccBlan]/_Jumping movement [Macc_MaccSalt]
	Wrist_Neutral position [Mca_McaN]/_Raised position [Mca_McaF]
	Forearm_ High [AtBz_AtBzA]
	Placement of the body in front of the keyboard_High [CC_CCa]

P2 Focal behaviors/elements	P2 Conditioned behaviors
Fingers with extension of the metacarpophalangeal joint and flexion of the proximal and distal interphalangeal joints. [DS_DextMfxPD]	Start of keystroke_Lifts finger before contact with the key [IPT_DelvextM]/_Arm lifted prior to the keystroke [IPT_Levprevbz]
	Fingers surface contact with the key_ Fingertip [DSct_Dcpt]/_Distal phalanx [DSct_DcfgD]/_Distal and proximal phalanx [DSct_DcfgDP]/_No contact [DSct_DNoc]
	Finger key release_ Lifting above the key [DLibT_Delv]
	Finger placement_Similar [DsCol_DsSim]/_ Disparate [DsCol_DsDisp]
	Hand: palm area_ Flat [MP_Mpl]
	Wrist_Neutral position [Mca_McaN]/_Raised position [Mca_McaF]/_Low position [Mca_McaEx]
	Forearm_ High [AtBz_AtBzA]/_ Low [AtBz_AtBzB]
	Placement of the body in front of the keyboard_High [CC_CCa]
Fingers with flexion of the metacarpophalangeal, proximal and distal joints. [DS_DfxMPD]	Start of keystroke_From contact with the key [IPT_Dcontsup]/_Lifts finger before contact with the key [IPT_ DelvextM]/_Arm lifted prior to the keystroke [IPT_Levprevbz]
	Fingers surface contact with the key_ Fingertip [DSct_Dcpt]/_ Fingerpad [DSct_Dcy]/_Distal phalanx [DSct_DcfgD]/_Distal and proximal phalanx [DSct_DcfgDP]/_No contact [DSct_DNoc]
	Finger key release_ Lifting above the key [DLibT_Delv]
	Finger placement_Similar [DsCol_DsSim]/_ Disparate [DsCol_DsDisp]
	Hand: palm area_ Flat [MP_Mpl]/_Cupped [MP_Moq]
	Wrist_Neutral position [Mca_McaN]/_Raised position [Mca_McaF]/_Low position [Mca_McaEx]
	Forearm_ High [AtBz_AtBzA]/_ Low [AtBz_AtBzB]
Thumb: surface contact via the lateral part of the thumb.[PScT_Pcl]	Thumb: movements_ pass with forearm rotation [P_PpsR]/_ pivots to allow passage of other fingers [P_PpivtPsDs]
	Hand: palm area_ Flat [MP_Mpl]
	Hand: action_ Movement with pronation turns [Macc_Mgirpro]/_ Movement with supination turns [Macc_Mgirsup]/_ White keys area [Macc_MaccBlan]/_ Back of keyboard [Macc_MaccFond]/_ Away from the keyboard [Macc_MaccFuer]/_Jumping movement [Macc_MaccSalt]
	Wrist_Neutral position [Mca_McaN]/_Raised position [Mca_McaF]/_Low position [Mca_McaEx]
	Forearm_High [AtBz_AtBzA]
	Placement of the body in front of the keyboard_High [CC_CCa]
9th demarcation Mozart Sonata N° 13 in B-flat major [ExMu_Mzt1_9]	Start of keystroke_Lifts finger before contact with the key [IPT_DelvextM]/_Arm lifted prior to the keystroke [IPT_Levprevbz]
	Fingers with flexion of the metacarpophalangeal joint and extension of the proximal and distal interphalangeal joints [DS_DfxMextPD]/_with extension of the metacarpophalangeal, distal and proximal joints [DS_DextMPD]
	Fingers surface contact with the key_Fingerpad [DSct_Dcy]/_Distal phalanx [DSct_DcfgD]/_No contact [DSct_DNoc]/_Fingertip [DSct_Dcpt]
	Thumb: surface contact_Lateral [PScT_PcL]/_Fingertip [PScT_PcP]/_No contact [PScT_PNoc]
	Finger: key release_ Lifting above the key [DLibT_Delv]
	Finger placement_Similar [DsCol_DsSim]/_ Disparate [DsCol_DsDisp]
	Hand: palm area_ Flat [MP_Mpl]
	Hand: action_ Back of keyboard [Macc_MaccFond]/_Jumping movement [Macc_MaccSalt]
	Wrist_Neutral position [Mca_McaN]/_Raised position [Mca_McaF]/_Low position [Mca_McaEx]
	Forearm_ High [AtBz_AtBzA]

Polar coordinate analysis allows us to obtain the prospective and retrospective Z_sum_ parameters of each conditioned behavior with respect to the focal behavior, and to visualize it via a vector map divided into four quadrants, where the prospective and retrospective Z_sum_ parameters are represented on the X and Y coordinate axes respectively, and the vector corresponding to each behavior is located in one of the four quadrants. This means there are four possibilities: quadrant I (++) indicates that the focal and conditioned behaviors are mutually activated; quadrant II (− +) indicates the prospective inhibition and retrospective activation of the behaviors, i.e., the focal behavior inhibits the conditioned behavior but is also activated by it; quadrant III (− −) indicates mutual prospective and retrospective inhibition; and quadrant IV (+ −) indicates prospective activation and retrospective inhibition, i.e., the focal behavior activates the conditioned behavior, although this inhibits the focal. The length of the vector (radius) is considered statistically significant (*p* < 0.05), when it measures from 1.96 onwards. The vector angle (angular coordinate) determines its location in one of the quadrants: I [0 to 90°]; II [91° to 180°]; III [181° to 270°]; IV [271° to 360°].

## 4 Results and discussion

### 4.1 Descriptive analysis

A total of 462 visualizations were carried out with participant P1, and 568 visualizations with P2, with the distribution by dimensions, behaviors and percentages of visualizations shown in Tables 3, 4, respectively.

**Table 3:** Record of visualizations by participant P1.

Dimensions	Behaviors	Visualization frequency	Percentage
Musical extract performed	1st demarcation	6	13.3
	2nd demarcation	3	6.7
	3rd demarcation	3	23.71
	4th demarcation	4	0.79
	5th demarcation	4	8.9
	6th demarcation	4	8.9
	7th demarcation	4	8.9
	8th demarcation	6	13.3
	9th demarcation	11	24.4
Start of keystroke	From contact with the key	21	100.0
	Lifts finger before contact with the key	0	0.0
	Lifts arm before keystroke	0	0.0
Fingers	Flexion M and slight extension P and D	21	37.5
	Flexion M, P and D	6	10.7
	Flexion M and extension P and D	17	30.4
	Extension M and flexion P and D	0	0.0
	Extension M, P and D	12	21.4
Fingers: surface contact with key	Fingerpad	28	60.9
	Distal phalanx	3	6.5
	Distal and proximal phalanx	2	4.3
	Fingertip	1	2.2
	Lateral in supination	0	0.0
	Lateral in pronation	0	0.0
	No contact	12	26.1
Thumb: surface contact	Lateral	25	56.8
	Fingerpad	1	1
	Fingertip	11	11
	No contact	7	15.9
Finger: key release	Sliding over the key	18	100.0
	Lifting over the key	0	0.0
Finger placement	Similar	45	100.0
	Disparate	0	0.0
Thumb: movements	Thumb pass with forearm rotation	0	0.0
	Thumb pivots to allow passage of other fingers	0	0.0
	Thumb slides to allow passage of other fingers	6	100.0
	Lateral thumb movement	0	0.0
Hand: palm area	Cupped	43	95.6
	Flat	2	4.4
Hand: action	Sliding movement	6	13.3
	Movement with pronation turns	0	0.0
	Movement with supination turns	1	2.0
	White keys area	37	82.2
	Back of keyboard	0	0.0
	Away from the keyboard	0	0.0
	Jumping movement	1	2.2
Wrist	Neutral position	7	15.6
	Lifted position	38	84.4
	Low position	0	0.0
Forearm	High	42	93.3
	Low	3	6.7
Placement of body in front of keyboard	High	45	100.0
	Low	0	0.0
	In line with the keyboard	0	0.0

**Table 4:** Record of visualizations by participant P2.

Dimensions	Behaviors	Visualization frequency	Percentage
Musical extract performed	1st demarcation	5	9.8
	2nd demarcation	3	5.9
	3rd demarcation	5	9.8
	4th demarcation	8	15.7
	5th demarcation	4	7.8
	6th demarcation	5	9.8
	7th demarcation	3	5.9
	8th demarcation	6	11.8
	9th demarcation	12	23.5
Start of keystroke	From contact with the key	4	14.8
	Lifts finger before contact with the key	13	48.1
	Lifts arm before keystroke	10	37.0
Fingers	Flexion M and slight extension P and D	1	1.4
	Flexion M, P and D	2	2.7
	Flexion M y extension P and D	2	2.7
	Extension M y Flexion P and D	39	53.4
	Extension M, P and D	29	39.7
Fingers: surface contact with key	Fingerpad	0	0.0
	Distal phalanx	3	5.3
	Distal and proximal phalanx	0	0.0
	Fingertip	25	43.9
	Lateral in supination	0	0.0
	Lateral in pronation	0	0.0
	No contact	29	50.9
Thumb: surface contact	Lateral	21	53.8
	Fingerpad	0	0.0
	Fingertip	7	17.9
	No contact	11	28.2
Finger: key release	Sliding over the key	1	10.0
	Lifting over the key	9	90.0
Finger placement	Similar	25	49.0
	Disparate	26	51.0
Thumb: movements	Thumb pass with forearm rotation	1	20.0
	Thumb pivots to allow passage of other fingers	4	80.0
	Thumb slides to allow passage of other fingers	0	0.0
	Lateral thumb movement	0	0.0
Hand: palm area	Cupped	3	5.9
	Flat	48	94.1
Hand: action	Sliding movement	0	0.0
	Movement with pronation turns	3	5.9
	Movement with supination turns	3	5.9
	White keys area	26	51.0
	Back of keyboard	11	21.6
	Away from the keyboard	1	2.0
	Jumping movement	7	13.7
Wrist	Neutral position	37	72.5
	Lifted position	13	25.5
	Low position	1	2.0
Forearm	High	36	70.6
	Low	15	29.4
Placement of body in front of keyboard	High	51	100.0
	Low	0	0.0
	In line with the keyboard	0	0.0

In line with Anguera (1982), corporal movements are non-verbal behaviors that occur quickly, can produce a large quantity of responses—each varying in frequency of occurrence—and can be present or absent in a given moment. Consequently, the frequency of occurrence measurement becomes relevant, given that it is possible to discover the action probability of a specific response and its comparison with other types of behavior. As the frequency data in Tables 3, 4 shows, there is a different set of motor behavior occurrences in the two participants.

**Figure 2:**
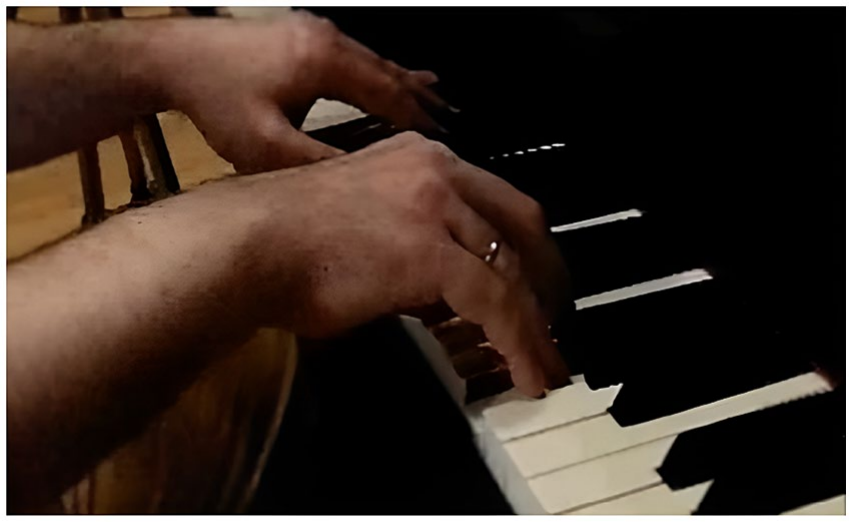
P1 Pressed touch: trajectory from distal phalanx to proximal phalanx.

With reference to participant P1, Figure 2 shows the middle finger pressing the “F” key, starting from contact with the key surface (behavior ITP_ Dcontsup achieved 100% of the visualizations), tracing a finger trajectory that goes from the DP to the PP (Furuya et al., 2010). The hand is cupped due to the marked lowering of the thumb and little finger and the flexion of the MJ (the behavior MP_Moq obtained 95.6% of the visualizations), it remains high due to the high position of the forearm (behavior AtBz_AtBzA obtained 93.3% of the visualizations), which lightens the weight avoiding the hand blocking, and all the fingers remain in contact with the keys (the behavior DsCol_DsSim obtained 100% of the visualizations).

**Figure 3:**
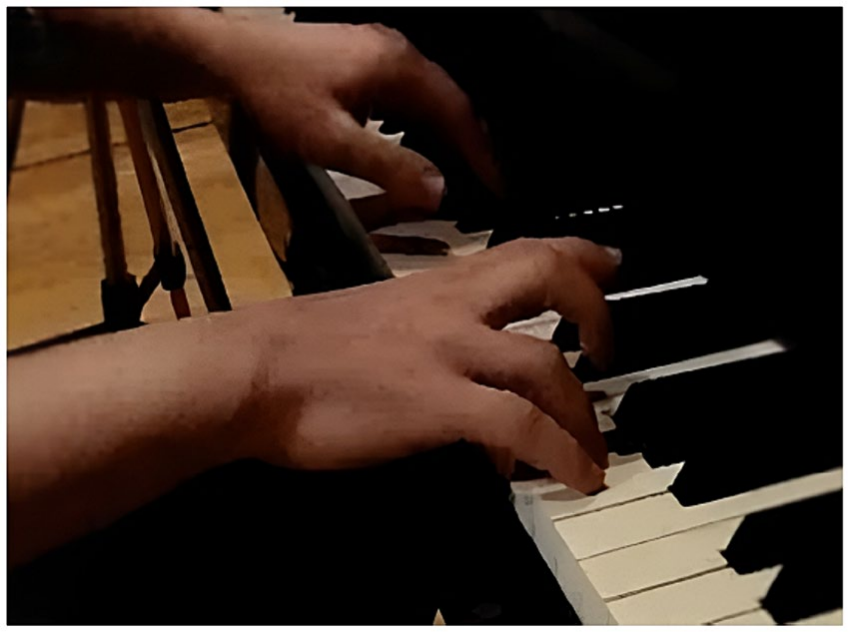
P2 Struck touch: trajectory from proximal phalanx to distal phalanx.

In the case of participant P2, Figure 3 shows prior finger elevation before pressing the “F” key, separating it from the key via extension of the MJ and flexion of DJ and PJ (the behavior IPT_DelvextM obtained 48.1% of the visualizations), tracing a finger trajectory that goes from PP to DP (Furuya et al., 2010). The hand is flat due to extension of the MJ and its position on the piano is low (the behavior MP_Mpl obtained 94.1% of the visualizations), due to a neutral wrist position (the behavior Mca_McaN obtained 72.5% of the visualizations) and the low position of the forearm (the behavior AtBz_AtBzB obtained 29.4% of the visualizations). The fingers are in disparate positions (observe the elevation of the index finger with the DJ and PJ extended and the middle finger with DJ and PJ flexed) (the behavior DsCol_DsDisp obtained 51% of the visualizations).

In keeping with the motor behavior patterns detailed above and those reflected in Tables 3, 4, two different types of touch are apparent—pressed and struck—which correspond to participant P1 and participant P2, respectively.

### 4.2 Polar coordinate analysis

The results of the polar coordinate analysis of the focal and conditioned behaviors shown in Table 2.

Participant P1. Motor interactions of the fingers that act with MJ flexed and PJ and DJ slightly extended (DS_DfxMligextPD) with the actions of start of keystroke (IPT), finger-key surface contact (DSct), key release actions (DlibT), finger placement (DsCol), shape of the palm area of the hand (MP), wrist position (Mca), forearm position (AtBz) and the placement of the body in front of the piano (CC).

**Figure 4:**
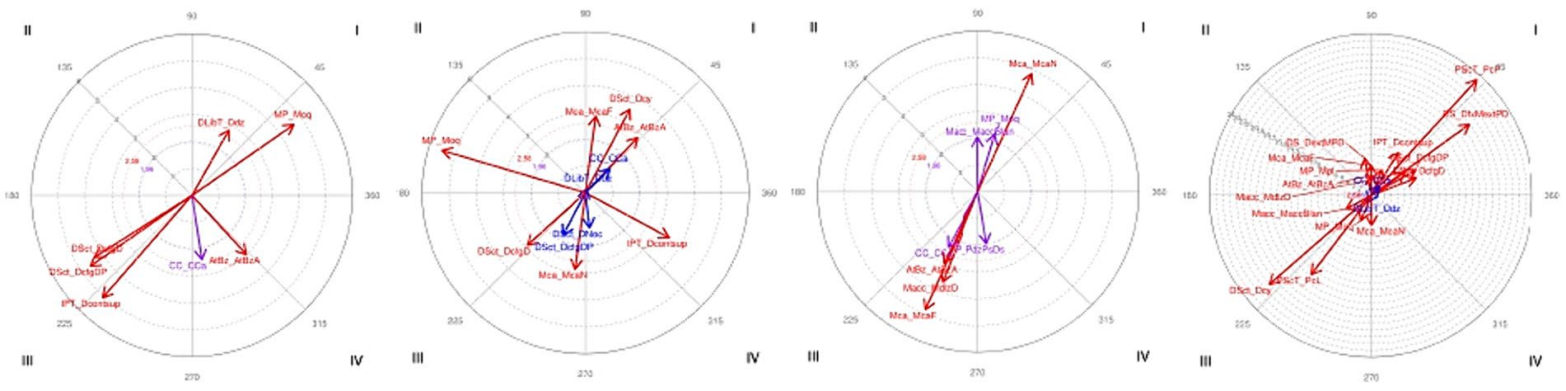
P1. Vector representation of the interactions between four given behaviors (focal) and their paired behaviors (conditioned). The panels from right to left correspond to the behaviors indicated in Table 2. Purple vectors represent significant interactions; red vectors represent highly significant interactions; and blue vectors represent slightly significant interactions (corresponding to the values marked with one or two asterisks, according to the significance level in Table 5).

The results of the analysis of the focal behavior DS_DfxMligextPD and the conditioned behaviors expressed in Table 2 for participant P1 were significant, as can be seen in block 1 of Table 5. In the corresponding vector map, shown first on the left in Figure 4, quadrant I shows the mutual activation of the focal behavior with the key release action of the fingers via sliding over it (DLibT_Ddz), along with a cupped palm shape (MP_Moq). The behavior relating to the finger-key surface contact is found in quadrant III, which means that it mutually inhibits the focal behavior, due to the moment of the action, prior to the key release via sliding. High forearm placement (AtBz_ AtBzA) is found in quadrant IV, which means that the fingers in MJ flexion and slight PJ and DJ extension activate the high forearm. At the same time the high forearm retrospectively inhibits the behavior of the fingers with flexed MJ and slightly extended PJ and DJ, which shows the rocking behavior of the forearm that acts in suspension, enabling the free movement of the fingers on regulating the pressure applied to them and avoiding the heaviness of the gravitational force (Ott, 2004). All this shows that the behavior of the fingers with MJ flexed and PJ and DJ slightly extended interacts with the cupped shape of the hand and the behavior of the high forearm. These behaviors facilitate key release via a sliding motion, motor behaviors that are characteristic of the pressed touch (Bellman, 2001; Furuya et al., 2012; Ortmann, 1925; Ott, 2003).

**Table 5:** Results of the polar coordinate analysis of participant P1.

Behavior	Quadrant	Prospective Zsum	Retrospective Zsum	Ratio	Radius	Significance	Angle
1. Focal behavior: fingers with flexion of the metacarpophalangeal joints and slight extension of the proximal and distal interphalangeal joints (DS_DfxMligextPD).							
IPT_Dcontsup	III	−3.35	−3.81	−0.75	5.07	**	228.72
DSct_Dcy	I	10.36	10.1	0.7	14.47	**	44.29
DSct_DcfgD	III	−3.65	−2.3	−0.53	4.32	**	212.22
DSct_DcfgDP	III	−3.81	−2.63	−0.57	4.63	**	214.66
DSct_DNoc	III	−8.23	−10.11	−0.78	13.04	**	230.86
DLibT_Ddz	I	1.38	2.44	0.87	2.8	**	60.42
MP_Moq	I	3.81	2.63	0.57	4.63	**	34.66
Mca_McaN	II	−1.72	6.77	0.97	6.98	**	104.26
Mca_McaF	IV	1.72	−6.77	−0.97	6.98	**	284.26
AtBz_AtBzA	IV	2.04	−2.2	−0.73	3	**	312.78
CC_CCa	IV	0.37	−2.4	−0.99	2.43	*	278.84
2. Focal behavior: fingers with flexion of the metacarpophalangeal, proximal and distal joints (DS_DfxMPD).							
IPT_Dcontsup	IV	3.07	−1.67	−0.48	3.5	**	331.4
DSct_Dcy	I	1.63	3.12	0.89	3.52	**	62.4
DSct_DcfgD	III	−2.14	−1.96	−0.68	2.9	**	222.55
DSct_DcfgDP	III	−0.82	−1.58	−0.89	1.78		242.47
DSct_DNoc	IV	0.16	−1.33	−0.99	1.33		276.77
DLibT_Ddz	I	0.05	0.16	0.96	0.17		73.14
MP_Moq	II	−5.27	1.58	0.29	5.5	**	163.35
Mca_McaN	III	−0.38	−2.85	−0.99	2.88	**	262.47
Mca_McaF	I	0.38	2.85	0.99	2.88	**	82.47
AtBz_AtBzA	I	1.92	2.06	0.73	2.81	**	47.07
CC_CCa	I	0.9	0.9	0.71	1.27		45
3. Focal behavior: lateral thumb surface contact (PScT_ PcL).							
P_PdzPsDs	IV	0.33	−1.94	−0.99	1.97	*	279.57
MP_Moq	I	0.64	2.16	0.96	2.25	*	73.6
Macc_MdlzD	III	−1.35	−3.38	−0.93	3.64	**	248.21
Macc_MaccBlan	II	0	2.04	1	2.04	*	90.14
Mca_McaN	I	2.01	442	0.91	4.85	**	65.56
Mca_McaF	III	−2.01	−4.42	−0.91	4.85	**	245.56
AtBz_AtBzA	III	−1.31	−2.69	−0.9	2.99	**	244.06
CC_CCa	III	−1.14	−2.08	−0.88	2.37	*	241.18
4. Focal behavior/elements: musical extract performed whole 9th bar two first beats of 10th bar (ExMu_ Mzt1_9).							
IPT_Dcontsup	I	4.04	6.34	0.84	7.52	**	57.49
DS_DfxMextPD	I	14.56	10.55	0.59	17.98	**	35.92
DS_DextMPD	II	−1.01	5.5	0.98	5.59	**	100.37
DSct_Dcy	III	−15.16	−13.24	−0.66	20.13	**	221.14
DSct_DcfgD	I	6.63	2.56	0.36	7.1	**	21.12
DSct_DcfgDP	I	6.58	3.75	0.5	7.58	**	29.67
PScT_PcL	III	−8.92	−11.78	−0.8	14.78	**	232.88
PScT_PcP	I	15.65	17.14	0.74	23.21	**	47.61
DLibT_Ddz	III	−0.54	−1.07	−0.89	1.2		243.24
MP_Moq	III	−1.58	−3.75	−0.92	4.07	**	247.12
MP_Mpl	I	1.58	3.75	0.92	4.07	**	67.12
Macc_MdlzD	I	5.03	3.45	0.56	6.1	**	34.4
Macc_MaccBlan	III	−3.73	−2	−0.47	4.23	**	208.26
Mca_McaN	IV	0.03	−4.44	−1	4.44	**	270.36
Mca_McaF	II	−0.03	4.44	1	4.44	**	90.36
AtBz_AtBzA	II	−0.02	3	1	3	**	90.38
CC_CCa	I	1.23	1.31	0.73	1.79		46.82

**Means very significant (< 0.01).

Participant P1. Motor interactions of the fingers that act with flexion of the MJ, PJ and DJ, (DS_DfxMPD), with the actions of start of keystroke (IPT), finger-key surface contact (DSct), key release action (DlibT), finger placement (DsCol), shape of the palm area of the hand (MP), wrist position (Mca), forearm position (AtBz) and the placement of the body in front of the piano (CC).

The results of the analysis of focal behavior DS_DfxMPD and the conditioned behaviors expressed in Table 2 for participant P1 were not significant in behaviors DSct_DcfgDP, DSct_DNoc, DLibT_Ddz and CC_CCa, as can be observed in block 2 of Table 5, while the rest of the results were significant. Second from the left in the corresponding vector map represented in Figure 4, clearly different motor behaviors and their location in opposing quadrants (activation–inhibition) are shown. In quadrant I the mutual activation of the focal behavior with the finger-key surface contact via the fingerpad, the wrist flexed and the forearm high is opposite to the behaviors in quadrant III, with the inhibition of the conditioned behavior relating to the finger-key contact with the DP and a neutral wrist position. In quadrant II we can see the shape of the palm area is cupped, indicating that the behavior of the fingers with MJ, PJ, and DJ flexed inhibits the cupped palm area prospectively, at the same time that the cupped hand retrospectively activates the finger action with the MJ, PJ, and DJ flexed. The action of the start of the keystroke from contact with its surface is located in quadrant IV, which means that the focal behavior (DS_DfxMPD) prospectively activates the conditioned behavior (IPT_Dcontsup), at the same time that the conditioned behavior retrospectively inhibits the focal. All this reveals that the motor behavior of the fingers with MJ, PJ and DJ flexed interacts with the finger-key contact of the fingerpad, the high forearm, and wrist flexion, which can be attributed to the fact that, in pressed keystrokes, the wrist anticipates the attack through wrist extension and steadily augments its contribution via wrist flexion (Oikawa et al., 2011; Goebl et al., 2005). Contact with the fingerpad provides greater tactile feedback, essential for spatial and temporal precision, along with the production of the pianist’s sound intentions (Dalla Bella and Palmer, 2011; Goebl and Palmer, 2008; James, 2012; Robb, 2022); these being motor behaviors characteristic of the pressed touch (Goebl et al., 2014; Furuya et al., 2010; MacRitchie, 2015; Verdugo et al., 2020).

Participant P1. Motor interactions of the action of thumb in lateral contact (PScT_PcL) with thumb actions (P), shape of the palm area of the hand (MP), hand action (Macc), wrist position (Mca), forearm position (AtBz) and placement of the body in front of the piano (CC).

**Figure 5:**
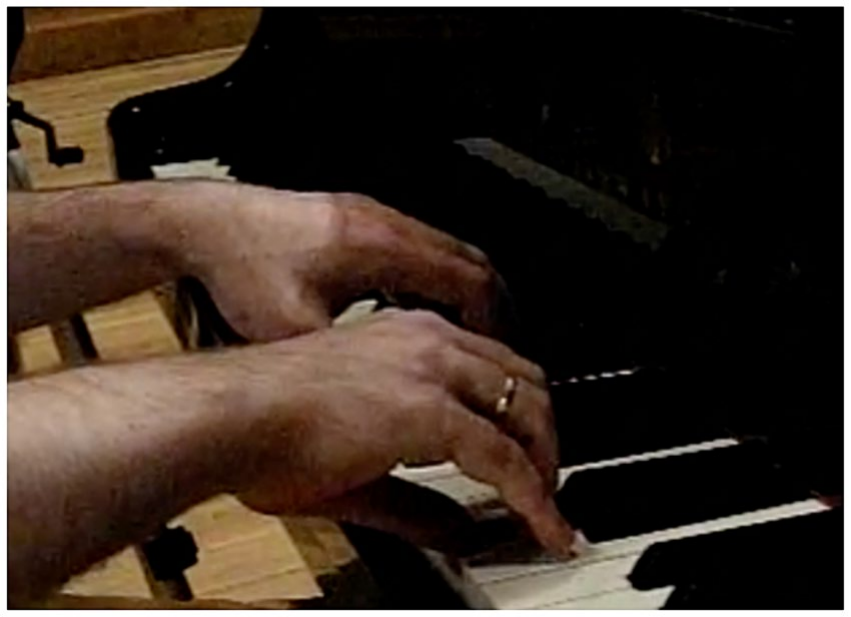
P1 Hand movement via thumb sliding.

The results of the analysis of focal behavior PScT_PcL and the conditioned behaviors expressed in Table 2 for participant P1 were significant, as can be seen in block 3 of Table 5. Third from the left in the corresponding vector map represented in Figure 4, we can see in quadrant I the mutual activation of the focal behavior with the neutral wrist position (Mca_McaN), together with mutual activation, although to a lesser degree but also significant, of the cupped hand shape (MP_Moq) and the action of the hand on the white keys (Macc_MaccBlan). Quadrant III shows the mutual inhibition of the conditioned behaviors relating to the high placement of the body in front of the keyboard (CC_CCa), high placement of the forearm (AtBz_AtBzA), movement of the hand via sliding (Macc_MdlzD) and a high wrist position (Mca_McaF). Quadrant IV shows the prospective activation of sliding the thumb over the key to enable the other fingers to pass over it, allowing lateral movement of the hand without this intervening, as is shown in quadrant III, whilst keeping its position in the white keys area and its cupped shape. Two clearly opposing activation actions are shown (neutral wrist, in quadrant I) and inhibition actions (flexed wrist, in quadrant III), due to the fact that the wrist does not use rotation or flexion for this movement. At the beginning of the sonata where there are six descending consecutive notes (G-F-E♭-D-C-B♭), P1 presses the “D” key with the thumb and allows the middle finger to press the “C” key by sliding over the “D” key. The hand does not change its placement or position, so that it moves laterally towards the left of the keyboard without a pronated turn. On the last beat of the second bar, the group of semiquavers (F-E♭-D-C) ends with the note “C,” on which the thumb carries out the same sliding action to allow the “B♭” at the beginning of the following bar to be played. The results show the interaction of the motor behavior of the thumb in lateral contact, with the thumb sliding over the key, the hand cupped, the hand moving via the thumb sliding over the key and the wrist in neutral position (see Figure 5).

Participant P1. Motor interactions of the action carried out in the musical extract of the 9th demarcation of the fragment of the Mozart sonata (ExMu_Mzt1_9) with the actions of start of keystroke (IPT), finger position (DS), finger-key surface contact (Dsct), thumb surface contact with the key (PScT), key release actions (DlibT), finger placement (DsCol), shape of the palm area of the hand (MP), hand action (Macc), wrist position (Mca), forearm position (AtBz) and the placement of the body in front of the piano (CC).

The results of the analysis of focal behavior ExMu_Mzt1_9 and the conditioned behaviors expressed in Table 2 for participant P1 were significant, as can be observed in block 4 of Table 5, with the exception of DLibT_Ddz and CC_CCa. Fourth from the left in the corresponding vector map represented in Figure 4, we can see in quadrant I the mutual activation of the focal behavior with the action of the thumb making contact with the tip and the placement of the fingers flexing MJ and extending PJ and DJ. The start of the keystroke with the rest of the fingers is done from contact of the DP and PP with the key surface; the hand is flat and moves via sliding the fingers without modifying its position (Macc_MdlzD). In quadrant II the behavior focal prospectively inhibits the conditioned behaviors of finger position with extension of MJ, PJ and DJ, the wrist flexed and the forearm high, at the same time that the conditioned behaviors retrospectively activate the action of the focal behavior. Quadrant III shows the mutual inhibition of the focal behavior and the conditioned behaviors relating to finger contact via the finger pad (DSct_Dcy), lateral thumb contact (PScT_PcL), cupped palm shape (MP_Moq) and the action of the hand over the white keys (Macc_Blan). Quadrant IV shows the prospective activation of the neutral wrist position, while this retrospectively inhibits the focal behavior.

**Figure 6:**
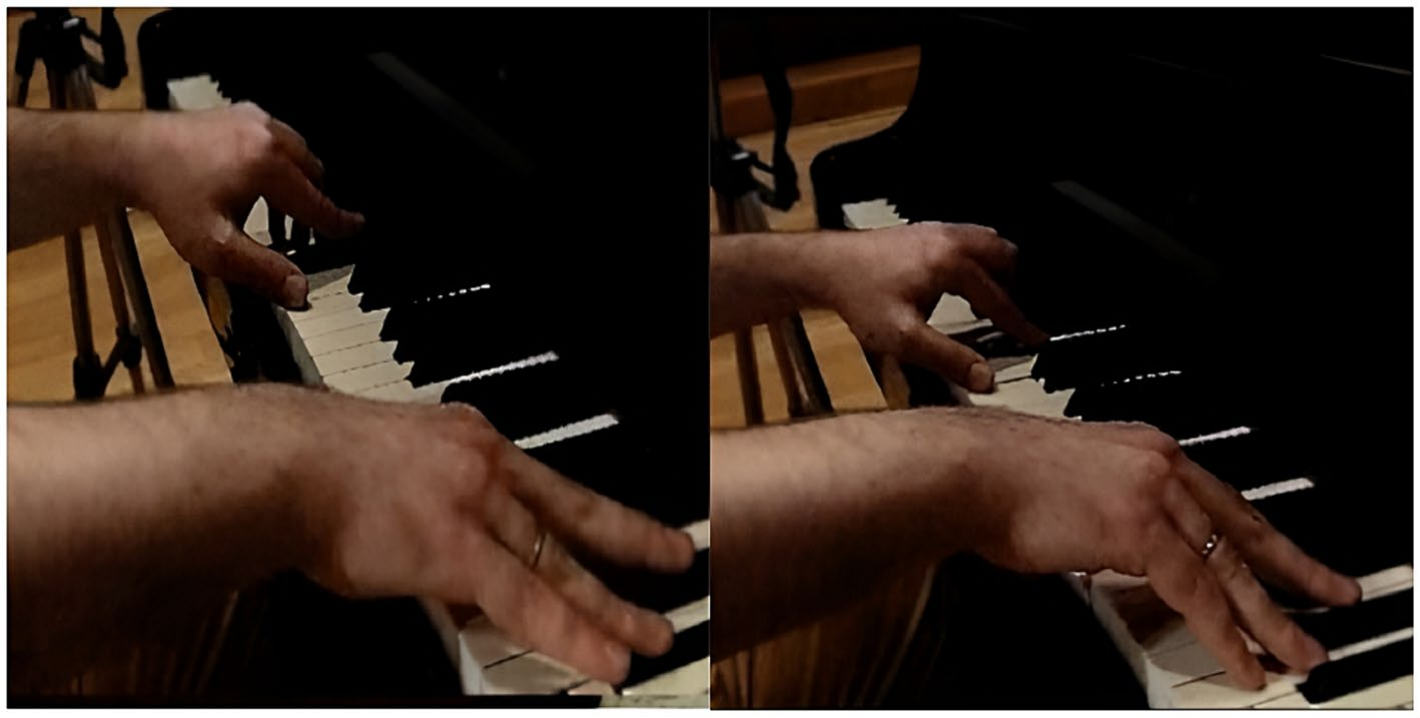
Movement of ring finger to little finger (“E-flat” – “D”) via sliding over the key.

In focal behavior ExMu_Mzt1_9 we can observe the actions produced in the whole of the 9th bar and the first two beats of the 10th bar of the sonata. At the start of the 9th bar there is a syncope that begins with an octave (F-F), in the strong part of the first beat. The tip of the thumb remains in contact throughout the whole syncope (F, minim figure) due to the distance maintained with the “F” key an octave higher and the following keys “E♭-D” and “D.” Here what is notable is the action of the ring finger sliding over the “E♭” key until the little finger presses the “D” key. This motor action is significant, considering that the hand is moving towards the left of the keyboard, without the ring finger moving to the middle, which is what is immediately to its left, but the ring finger slides over the key to allow the little finger to move, which is on its right, an action which enables the sound continuity of the syncope (Bellman, 2001; Ott, 2003). Finally, a succession of descending sixths is played by sliding the fingers over the keys. The results show the sliding action of the fingers which interact with the rest of the motor behaviors (see Figure 6).

Participant P2. Motor interactions of the fingers that act with extension of the MJ and flexion of the PJ and DJ (DS_DextMfxPD) with the actions of start of keystroke (IPT), finger-key surface contact (DSct), key release actions (DlibT), finger placement (DsCol), shape of the palm area of the hand (MP), wrist position (Mca), forearm position (AtBz) and the placement of the body in front of the piano (CC).

**Figure 7:**
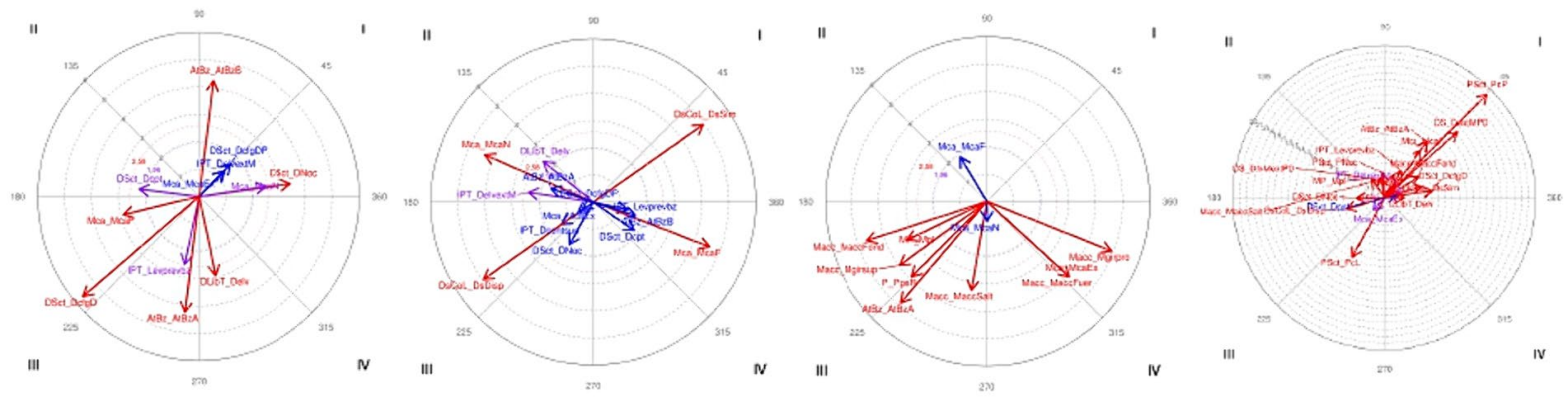
P2. Vector representation of the interactions between four given behaviors (focal) and their paired behaviors (conditioned). The panels from right to left correspond to the behaviors indicated in Table 2. Purple vectors represent significant interactions; red vectors represent highly significant interactions; and blue vectors represent slightly significant interactions (corresponding to the values marked with one or two asterisks, according to the significance level in Table 6).

The results of the analysis of focal behavior DS_DextMfxPD and the conditioned behaviors expressed in Table 2 for participant P2 were significant, with the exception of IPT_DelvextM, DSct_DcfgDP and Mca_McaEx, as shown in block 1 of Table 6. First on the left in the corresponding vector map represented in Figure 7, we can see the behaviors with the longest vectors in each of the quadrants. Thus, quadrant I shows the mutual activation of the focal behavior with the conditioned behaviors of no finger-key contact (DSct_DNoc), neutral wrist position (Mca_McaN) and low forearm (AtBz_AtBzB). Quadrant III shows the mutual inhibition of the behaviors relating to the start of the keystroke with prior lifting of the arm, along with finger-key contact with the DP, flexed wrist and high forearm. Quadrant II shows the prospective inhibition of the conditioned behavior relating to finger-key contact via the fingertip (DSct_Dcpt), at the same time that this retrospectively activates the action of the fingers with extension of the MJ, and flexion of the PJ and DJ. The key release action via lifting the finger is located in quadrant IV, which means that the focal behavior (DS_DextMfxPD) prospectively activates the conditioned behavior (DLibT_Delv), while the conditioned behavior inhibits the focal retrospectively. These interactions are related to the flattening of the hand arch that occurs with the given focal behavior, necessitating the raising of the fingers or forearm to release or touch the key due to the tension created in the forearm and fingers (Brown, 2000).

**Table 6:** Results of the polar coordinate analysis of participant P2.

Behavior	Quadrant	Prospective Zsum	Retrospective Zsum	Ratio	Radius	Significance	Angle
1. Focal behavior: fingers with extensión of the metacarpophalangeal joints and flexion of the proximal and distal interphalangeal joints (DS_ DextMfxPD).							
IPT_DelvextM	I	0.94	0.92	0.7	1.32		44.46
IPT_Levprevbz	III	−0.54	−2.48	−0.98	2.54	*	257.64
DSct_Dcpt	II	−2.24	0.27	0.12	2.25	*	173.18
DSct_DcfgD	III	−4.32	−3.65	−0.65	5.66	**	220.21
DSct_DcfgDP	I	1.17	1.19	0.71	1.66		45.52
DSct_DNoc	I	3.44	0.46	0.13	3.47	**	7.68
DLibT_Delv	IV	0.61	−2.89	−0.98	2.95	**	281.95
DsCoL_DsSim	III	−5.3	−4.5	−0.65	6.96	**	220.33
DsCoL_DsDisp	I	5.3	4.5	0.65	6.96	**	40.33
MP_Mpl	I	6.04	1.75	0.28	6.29	**	16.18
Mca_McaN	I	2.48	0.36	0.14	2.5	*	8.25
Mca_McaF	III	−2.83	−0.66	−0.23	2.91	**	193.2
Mca_McaEx	I	0.9	0.93	0.72	1.29		45.96
AtBz_AtBzA	III	−0.53	−4.22	−0.99	4.26	**	262.85
AtBz_AtBzB	I	0.53	4.22	0.99	4.26	**	82.85
2. Focal behavior: fingers with flexion of the metacarpophalangeal, proximal, and distal joints (DS_DfxMPD).							
IPT_Dcontsup	III	−1.17	−0.82	−0.58	1.43		215.13
IPT_DelvextM	II	−2.41	0.35	0.15	2.43	*	171.65
IPT_Levprevbz	IV	1.27	−0.28	−0.22	1.3		347.49
DSct_Dcpt	IV	1.51	−1.05	−0.57	1.84		325.18
DSct_DcfgD	II	−0.91	8.14	0.99	8.19	**	96.36
DSct_DcfgDP	III	−0.4	−0.28	−0.58	0.49		215.16
DSct_DNoc	III	−0.88	−1.57	−0.87	1.8		240.67
DLibT_Delv	II	−1.85	1.5	0.63	2.38	*	141.1
DsCoL_DsSim	I	4.06	2.85	0.58	4.96	**	35.1
DsCoL_DsDisp	III	−4.06	−2.85	−0.58	4.96	**	215.1
MP_Mpl	III	−10.6	−3.24	−0.29	11.08	**	197.02
MP_Moq	I	10.6	3.24	0.29	11.08	**	17.02
Mca_McaN	II	−4.01	1.74	0.4	4.37	**	156.57
Mca_McaF	IV	4.29	−1.65	−0.36	4.6	**	338.95
Mca_McaEx	III	−0.56	−0.4	−0.58	0.69		215.14
AtBz_AtBzA	II	−1.56	0.5	0.3	1.64		162.27
AtBz_AtBzB	IV	1.56	−0.5	−0.3	1.64		342.27
3. Focal behavior: thumb contact surface on the lateral side (PScT_ PcL).							
P_PpsR	III	−2.78	−2.74	−0.7	3.9	**	224.64
P_PpivtPsDs	IV	5.59	−3	−0.47	6.34	**	331.81
MP_Mpl	III	−2.94	−1.4	−0.43	3.26	**	205.5
Macc_Mgirpro	IV	4.6	−1.81	−0.37	4.94	**	338.56
Macc_Mgirsup	III	−3.22	−2.33	−0.59	3.97	**	215.88
Macc_MaccBlan	I	2.45	6.15	0.93	6.62	**	68.23
Macc_MaccFond	III	−4.44	−1.43	−0.31	4.66	**	197.88
Macc_MaccFuer	IV	3.03	−2.74	−0.67	4.09	**	317.87
Macc_MaccSalt	III	−0.57	−3.24	−0.98	3.29	**	259.98
Mca_McaN	IV	0.01	−0.73	−1	0.73		270.7
Mca_McaF	II	−0.98	1.63	0.86	1.9		121.19
Mca_McaEx	IV	3.03	−2.74	−0.67	4.09	**	317.87
AtBz_AtBzA	III	−3.18	−3.7	−0.76	4.88	**	229.3
4. Focal behavior/elements: musical extract performed whole 9^th^ bar two first beats of 10th bar (ExMu_ Mzt1_9).							
IPT_DelvextM	I	2.44	0.71	0.28	2.54	*	16.16
IPT_Levprevbz	I	2.37	3.85	0.85	4.52	**	58.42
DS_DfxMextPD	II	−1.9	2.74	0.82	3.34	**	124.74
DS_DextMPD	I	10.71	9.45	0.66	14.28	**	41.43
DSct_DcfgD	I	7.14	0.97	0.14	7.21	**	7.77
DSct_DNoc	III	−4.11	−0.07	−0.02	4.11	**	181
DSct_Dcpt	I	1.68	0.33	0.2	1.71		11.28
PSct_PcL	III	−4.73	−8.61	−0.88	9.82	**	241.2
PSct_PcP	I	15.02	14.95	0.71	21.19	**	44.86
PSct_PNoc	II	−0.33	3.59	1	3.61	**	95.2
DLibT_Delv	IV	2.82	−0.04	−0.01	2.82	**	359.21
DsCoL_DsSim	I	5.54	1.64	0.28	5.78	**	16.48
DsCoL_DsDisp	III	−5.54	−1.64	−0.28	5.78	**	196.48
MP_Mpl	II	−0.64	2.9	0.98	2.97	**	102.4
Macc_MaccFond	I	5.07	3.06	0.52	5.92	**	31.06
Macc_MaccSalt	I	1.31	3.17	0.92	3.43	**	67.54
Mca_McaF	I	5.39	6.79	0.78	8.67	**	51.55
Mca_McaEx	III	−1.71	−1.81	−0.73	2.49	*	226.64
AtBz_AtBzA	I	6.34	8.1	0.79	10.29	**	51.94

**Means very significant (< 0.01).

**Figure 8:**
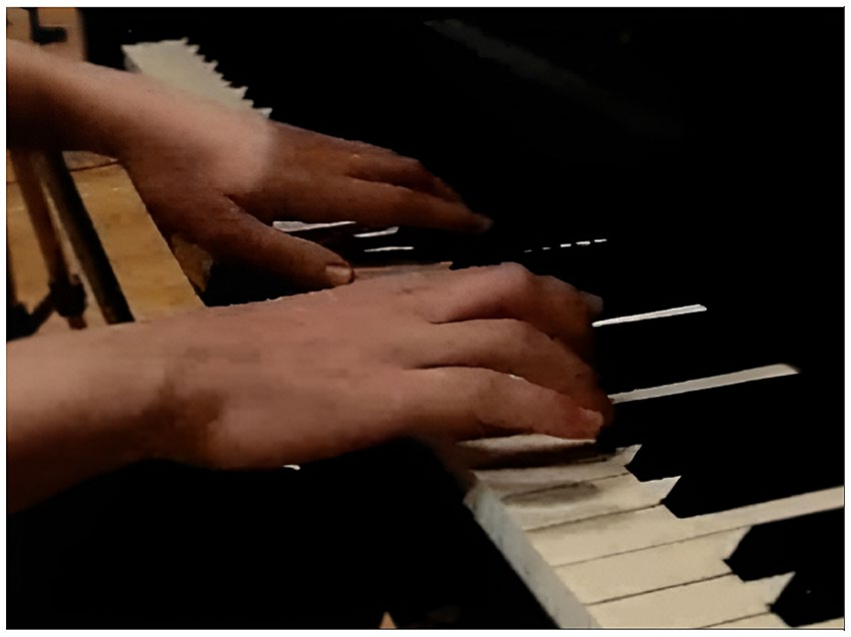
P2 Key release via finger elevation and start of keystroke from a certain distance from the keys.

The results show the interaction of the motor behavior of fingers with extended MJ and flexed PJ and DJ, with fingertip finger-key contact (Berman, 2002), and key release via lifting the finger and the start of the keystroke from a certain distance from the keys, via the action of no finger-key contact (Furuya et al., 2010); these being behaviors characteristic of the motor behavior of the struck touch (see Figure 8).

Participant P2. Motor interactions of the fingers that act with flexion of the MJ, PJ and DJ, (DS_DfxMPD), with the actions of start of keystroke (IPT), finger-key surface contact (DSct), key release actions (DlibT), finger placement (DsCol), shape of the palm area of the hand (MP), wrist position (Mca), and forearm position (AtBz).

The results of the analysis of focal behavior DS_DfxMPD and the conditioned behaviors expressed in Table 2 for participant P2 were significant, with the exception of behaviors IPT_Dcontsup, IPT_Levprevbz, DSct_Dcpt, DSct_DcfgDP, DSct_DNoc, Mca_McaEx, AtBz_AtBzA and AtBz_AtBzB, which were not significant, as can be seen in block 2 of Table 6. Second from the left in the corresponding vector map, represented in Figure 7, we can see the behaviors with the longest vectors in each of the quadrants. Thus, quadrant I shows the mutual activation of the focal behavior with a similar finger placement (DsCol_DsSim), a behavior opposite to that shown in quadrant III, relating to the inhibition of disparate finger placement (DsCol_DsDisp). Quadrant II shows a flat palm area (MP_Mpl), indicating that the focal behavior prospectively inhibits the conditioned behavior (MP_Mpl), at the same time that the conditioned behavior—i.e., the flat hand—retrospectively activates the focal behavior, that is, the action of the fingers with flexion of the MJ, PJ, and DJ. The flexed wrist position (Mca_McaF) is located in quadrant IV, as opposed to the neutral wrist position (Mca_McaN) of quadrant II, which means that the focal behavior (DS_DfxMPD) prospectively activates the conditioned behavior (Mca_McaF), at the same time that the conditioned behavior inhibits the focal, just the opposite as to what happens in quadrant II (Mca_McaN). This motor interaction of the wrist can be attributed to the fact that, in struck keystrokes, wrist flexion assists the downward movement of the fingertip prior to the attack. Its contribution diminishes upon finger-key contact and increases towards the end of the attack (Verdugo et al., 2020). Quadrant IV also shows the prospective action of lifting the arm prior to the start of the keystroke (IPT_Levprevbz), along with finger-key contact via the fingertip (DSct_Dcpt), while in quadrant III it inhibits—both prospectively and retrospectively—the behavior that characterizes the pressed touch, i.e., the start of the keystroke from contact with the surface of the key (IPT_Dcontsup).

The results show the interaction of the motor behavior of fingers with flexions MJ, PJ and DJ with a similar finger placement, a flat hand, prior elevation of the arm before the start of the keystroke, and finger-key contact via the fingertip, which are motor behaviors associated with the struck touch (Goebl et al., 2014; Furuya et al., 2010; Verdugo et al., 2020).

Participant P2. Motor interactions of the action of thumb in lateral contact (PScT_PcL) with thumb actions (P), shape of the palm area of the hand (MP), hand action (Macc), wrist position (Mca), forearm position (AtBz) and placement of the body in front of the piano (CC).

The results of the analysis of focal behavior PScT_PcL and the conditioned behaviors expressed in Table 2 for participant P2 were significant, with the exception of behaviors Mca_McaN and Mca_McaF, that were not significant, as can be observed in block 3 of Table 6. Third from the left in the corresponding vector map represented in Figure 7, we can see the behaviors with the longest vectors in each of the quadrants. Quadrant I shows the mutual activation of the hand action in the white keys area. This behavior is not visible in the vector map due to the long length of the vector (6.62), as can be seen in Table 6. Quadrant III shows the inhibition of the conditioned behaviors Macc_MaccFond, MP_Mpl, Macc_Mgirsup, P_PpsR, AtBz_AtBzA and Macc_MaccSalt. Quadrant IV shows the prospective activation of the hand action with pronated turn (Macc_Mgirpro), the thumb acting as a pivot to allow the movement of the other fingers (P_ PpivtPsDs), visible in Table 6 by the long length of its vector (6.34), hand action with the palm away from the keyboard (Macc_MaccFuer) and wrist lowered, via joint extension (Mca_McaEx), while the conditioned behavior inhibits the focal behavior. In movements carried out in the succession of descending sounds by consecutive degrees, the thumb remains fixed on the key and turns in pronation which the hand follows to reposition itself after the fingers have passed. The thumb releases the key via a lifting of the finger, when the rest of the fingers press the next key. The results show the interaction of the lateral thumb contact behavior with motor behaviors related to rotation of the thumb and the hand, together with thumb key release via elevation (Goebl et al., 2005).

Participant P2. Motor interactions of the action carried out in the musical extract of the 9th demarcation of the fragment of the Mozart sonata (ExMu_Mzt1_9) with the actions of start of keystroke (IPT), finger position (DS), finger-key surface contact (Dsct), thumb surface contact with the key (PScT), key release actions (DlibT), finger placement (DsCol), shape of the palm area of the hand (MP), hand action (Macc), wrist position (Mca), forearm position (AtBz) and the placement of the body in front of the piano (CC).

The results of the analysis of focal behavior ExMu_Mzt1_9 and the conditioned behaviors expressed in Table 2 for participant P2 were significant, with the exception of DSct_Dcpt, which were not significant, as can be seen in block 4 of Table 6. Fourth from the left in the corresponding vector map represented in Figure 7, we can see the behaviors with the longest vectors in each of the quadrants, where almost all the behaviors are presented in quadrants I and III, which are opposites (mutual activation and mutual inhibition). Thus, quadrant I shows the motor behavior of the fingers with extension of MJ, PJ and DJ (DS_DextMPD), the start of the keystroke via prior lifting of the fingers with extension of MJ (IPT_DelvextM), start of keystroke via prior lifting of the arm (IPT_Levprevbz), the action of thumb contact via the tip (PScT_PcP), the surface contact of the rest of the fingers with the DP (DSct_DcfgD), similar finger placement (DsCol_DsSim), hand action at the back of the keyboard (Macc_MaccFond), jumping hand movement (Macc_MaccSalt), flexed wrist position (Mca_McaF) and high forearm (AtBz_AtBzA). Quadrant II shows that the focal behavior inhibits the conditioned behaviors of finger position with MJ flexion and PJ and DJ extension (DS_DfxMextPD), thumb not in contact with the key surface (PScT_PNoc) and flat palm area (MP_Mpl), at the same time that the conditioned behaviors activate the focal behavior action. Quadrant III shows the inhibition of the conditioned behaviors relating to the action of the fingers not in contact with the surface of the keys (DSct_DNoc), lateral thumb contact (PSct_PcL), disparate finger placement (DsCol_DsDisp) and wrist position with joint extension (Mca_McaEx). Quadrant IV shows the prospective activation of the key release action via lifting the fingers over the keys (DLibT_Delv), while the conditioned behavior inhibits the focal behavior. As previously outlined, in the focal behavior ExMu_Mzt1_9 we can observe the motor actions involved in the playing of the syncope, where we can see the thumb tip in contact with the key until the end of the syncope. The forearm moves forward to press the “D” note of the syncope by placing the finger on the narrow part of the white key, between the two black keys, with the hand acting at the back of the keyboard. This is followed by a succession of descending sixths which are played via jumping hand movements to move from one sixth to the next. The keys are released by lifting the hand, which produces a sound discontinuation in the passage from some sounds to others.

The results show the interaction of the focal behavior with the motor behaviors of the fingers with extension MJ, PJ and DJ, thumb tip contact with the key, hand action at the back of the keyboard, with jumping hand movements, a flexed wrist position and key release via lifting the fingers and the forearm.

The results obtained with respect to frequency in the scrutiny of motor behavior occurrences produced by participants P1 and P2 clearly correspond with the polar coordinate analysis of the motor behaviors that characterize the pressed touch and the struck touch, respectively.

## 5 Conclusion

The aim of this study was to detect and analyze the interactions of motor behaviors that distinctively characterize pressed and struck touch in piano performance. Additionally, it aimed to show the huge potential offered by research into motor behavior in piano playing from the scientific focus of mixed methods.

Systematic observation is an ideal medium for defining the piano touch procedure and for interpreting the expressive significance of body movement, which goes beyond anatomical and biomechanical knowledge (Goebl, 2017). Observational methodology, which brings a novel perspective to artistic research (Anguera, 2010). The possibility of using computer applications both in the musical research and professional fields, opens up a pathway of innovation and improvement that unites research and practice, implying an effective transfer of knowledge and therefore the evolution towards refinement in the field of musical performance.

Lag sequential analysis was used to examine the strength of association between behaviors using HOISAN software. Polar coordinate analysis, based on adjusted residuals from lag sequential analysis, identified activating or inhibitory relationships between a behavior of interest, known as a focal behavior, and other behaviors, known as conditioned behaviors.

Through observational methodology, this study has extracted relevant defining patterns of motor behavior in piano touch procedure. The findings reveal novel interactions between motor behaviors associated with two types of touch, contributing to the existing literature on motor control in piano performance.

Thus, in the pressed touch, the flexion of the metacarpophalangeal joints (knuckles) and slight extension of the P and D joints interact with the cupped hand and the finger-key contact surface centered on the finger pads. The contact surface of the fleshy part of the finger is larger, which allows for more precise control during performance (Dalla Bella and Palmer, 2011; Goebl and Palmer, 2008; James, 2012; Robb, 2022). The cupped position of the hand interacts with the key release behavior through the sliding of the fingers, which in turn interacts with the raised forearm, facilitating the cupping of the hand while avoiding the heaviness of the arm weight by keeping it in suspension, resulting in an improvement in sound quality. The study has highlighted the importance of sliding the fingers over the keys towards the palms of the hands in a circular, chained motion that goes from the DP to the PP, and in the case of the thumb, its sliding facilitates the passage of the other fingers. Pressure comes from contact made with the keys before they are pressed. The position of the hand is high, with slightly curved fingers in alignment (Del Pueyo, 1990). The movement is controlled by suspension of the forearm, enabling sound continuity. The position of the hand is not altered by the movements over the keyboard thanks to the sliding motion of the fingers over the keys (Lee, 2005; Ott, 2004). The hand is close to the keyboard, which avoids the noise produced by the contact of the fingers with the keys. At the same time a greater economy of movement is produced since there are no big movements (Goebl et al., 2005).

In the struck touch, the extension of the metacarpophalangeal joints with flexion of the P and D joints interacts with the flat shape of the hand and the finger-key contact surface centered on the fingertip. The keystroke begins with the finger at a certain distance from the key, raised vertically via extension of the MJ before the key is pressed, going from the PP to the DP, or via elevation of the forearm, producing a noise upon contact that affects sound quality. Wrist activity is increased to stabilize the impact of the key’s inertia when struck (Verdugo et al., 2020). The finger releases the key vertically, via extension of the MJ or via elevation of the forearm, reducing the time it stays on it (Furuya et al., 2010; Goebl et al., 2014). The key makes contact with the fingertip, the contact surface of which is smaller and so, therefore, is the tactile feedback about sound control (Goebl et al., 2014). Movements across the keyboard are made via thumb, hand and forearm turns. These changes of position alter the sound continuity (Goebl et al., 2005).

The described behavioral interactions were also supported by the descriptive analysis of observed behaviors in both pianists, measured by occurrence frequency, which is relevant in body movements (Anguera, 1982). Through the percentage of response visualization, the most significant behaviors were evidenced in both participants, highlighting behaviors associated with two types of touch, pressed and struck, corresponding to participants P1 and P2, respectively.

The results obtained in this study using observational methodology have successfully addressed the established aims, delving into the analysis of the right upper limb movement in the participating professional pianists. Furthermore, the study introduces new perspectives for supporting piano teachers, showing the effectiveness of systematic observation of pianist motor behavior. Future studies could be based on a bigger sample, along with the direct observation of the whole kinematic chain of piano-playing motion.

## Data Availability

The original contributions presented in the study are included in the article/supplementary material, further inquiries can be directed to the corresponding author.
